# Optimization of Cutting Data and Tool Inclination Angles During Hard Milling with CBN Tools, Based on Force Predictions and Surface Roughness Measurements

**DOI:** 10.3390/ma13051109

**Published:** 2020-03-02

**Authors:** Andrzej Matras, Wojciech Zębala

**Affiliations:** Production Engineering Institute, Mechanical Faculty, Cracow University of Technology, Al. Jana Pawła II 37, 31-864 Kraków, Poland; zebala@mech.pk.edu.pl

**Keywords:** optimization, cutting strategy, hard milling, free-form surface milling, CBN tools

## Abstract

This work deals with technological considerations required to optimize the cutting data and tool path pattern for finishing the milling of free-form surfaces made of steel in a hardened state. In terms of technological considerations, factors such as feed rate, workpiece geometry, tool inclination angles (lead and tilt angles) and surface roughness are taken into account. The proposed method is based on calculations of the cutting force components and surface roughness measurements. A case study presented in the paper is based on the AISI H13 steel, with hardness 50 HRC and milling with a cubic boron nitride (CBN) tool. The results of the research showed that by modifications of the feed value based on the currently machined cross-sectional area, it is possible to control the cutting force components and surface roughness. During the process optimization, the 9% and 15% increase in the machining process efficiency and the required surface roughness were obtained according to the tool inclination angle and feed rate optimization procedure, respectively.

## 1. Introduction

Cubic boron nitride (CBN) is an extremely hard material with excellent physical and chemical properties such as heat resistance, high modulus and weak reaction of other elements [1]. CBN, unlike diamond and due to the lower reactivity with the iron, is suitable for the machining of steel [2]. So, besides the grinding and electrical discharge machining process (EDM), cutting tools made of CBN are suitable in the production of molds and dies in the hard state, in precise and very precise operations of roughing [3,4,5,6].

Many authors have published their research relating to the machinability of hard materials using tools from the CBN. Matras et al. [7] machined a part of a spherical surface by using a ball nose cutter with CBN edges. The three various milling workpieces were analyzed (with hardnesses of 50, 62 and 65 HRC). Okada et al. [8] machined the steel with a hardness of 60 HRC with high cutting speed *v_c_*, reaching up to 600 m/min by using CBN tools. Aslan [9] obtained a surface with low surface roughness, Ra = 0.32 µm, and high tool life. Wojciechowski et al. [10] proposed a method for the reduction of cutting forces and the improvement of finish ball end milling efficiency of hardened steel 55NiCrMoV6.

Currently, many researchers perform their experiments for the selection of a technology and production system. The authors of publications very often propose computer systems for the selection of process technology. They are based on criteria related to the workpiece geometry, material hardness, surface roughness and dimensional-shape accuracy. Klocke et al. [11] noted that the grinding is suitable only for the production of parts with a relatively simple geometry. It is possible to use with materials with a hardness of more than 60 HRC, and enables a low surface roughness of workpiece after machining. The EDM process is suitable for the treatment of complex geometry parts made of conducting materials, regardless of their hardness. Navas et al. [12] reported its high dimensional accuracy and low surface roughness, but it is characterized by low productivity, which is a major problem in its application. However, high speed cutting (HSC) machining conducted with the usage of CBN milling tools is suitable for machining of hard materials with complex geometries, including those based on free-form surfaces with low surface roughness, high dimensional precision and high machining efficiency [13]. 

Milling of free-form surfaces is an expensive process. In order to improve the efficiency of free-form surfaces milling, many researchers deal with the optimization of this process. The works presented by them concern the application of numerical modeling for the process parameters’ optimization. Beňo at al. [14] analyzed the main features of ball end milling, and proposed a sequence of steps to identify the most suitable milling strategy.

Zębala and Plaza [15] proposed a method that minimizes the costs of the free-form surfaces’ milling. The presented application of the machining process optimization gives the opportunity to reduce the surface roughness parameters almost twofold and to shorten the machining time by approximately 17%. In turn, Durakbasa et al. [16] optimized the end milling process parameters for the surface quality of AISI H13 steel by using the Taguchi method. They determined the effect of the feed rate, cutting speed, tool tip radius and tool coating type on surface roughness. Chen et al. [17] machined a flat surface made of steel H13. They investigated only the effect of the tool inclination on the surface roughness. Similarly, Gao et al. [18] investigated the effect of a spherical cutter inclination on the shape and surface roughness of a microgroove made on a flat surface. They demonstrated that the use of an appropriate tool tilting strategy can significantly reduce surface roughness and improve the shape of the groove. 

Bouzakis et al. [19] proposed a method for optimizing the multi-axis machining of free-form surfaces. Based on the required surface roughness, they optimized the angle and direction of the tool inclination, radial depth of the cut and the feed speed. In their research, they did not take into account the variability of the shape of the surface being machined. In turn, Ikua et al. [20] developed a mathematical model that allows the calculation of machining error and the values of the cutting force components during the milling of concave and convex surfaces with spherical cutters. However, they analyzed only a few selected surface shapes. Scandiffio et al. [21] analyzed the effect of the tool inclination angle, not only on the surface roughness and values of cutting force components, but also tool wear. During the tests, they worked on a fragment of a circular convex surface made of D6 steel with a hardness of 60HRC. Without analyzing the variability of the surface’s shape to be machined, they determined the optimal value of the tool inclination angle. They also demonstrated the need to develop a method that allows this angle to be changed depending on the inclination angle of the workpiece.

Ozturk et al. [22] studied the influence of the cutting tool inclination on the surface roughness and the values of the cutting forces. Theoretical calculations were verified by means of the experiment. It was observed that the lead angle should be kept at a slightly positive value, since application of higher values of this angle shifts the engagement region to the upper parts of the cutting tool. Negative lead angles may result in tool tip contact. However, the authors [17] determined that for milling H13 steel with cemented carbide ball nose cutters, the optimal value for the tilt angle is from 40° to 20° for pull milling, or 20° to 40° for push milling, and for the lead angle it is approximately 25° for oblique revers milling. Matras et al. [7] applied ball nose cutters with edges made from CBN for milling hardened steel with an optimal lead angle equal to 8° and oblique revers milling, for a constant tilt angle equal to 0°_._ Yao et al. [23] also investigated the effect of the inclination angle of the tool and surface shape on its roughness. They analyzed four different surface shapes that machined with the “Zig” strategy. They stated that both the shape of the surface and the direction and angle of the tool influenced the surface roughness. The machining should be carried out in such a way as to maintain a constant angle of the tool inclination relative to the surface. 

Saffar et al. [24] used the finite element method (FEM) to calculate the components of the cutting force and tool deformation. Material properties were defined based on the Johnson–Cook theory. They used the deformation functions, strain rate and temperature of the workpiece, which allowed better material definition than in the form of constant values of coefficients, which takes place in the case of theoretical calculations. The usage of simulation based on the FEM also gave the possibility of defining the non-linear shapes of semi-finished products. They obtained a good match between simulation and experiment results, which confirmed the usefulness of the proposed method.

The purpose of this paper, regarding the complexity of machining of parts having free-form surfaces made from hardened materials, is a description of a new optimization method developed by the authors. It allows one to modify the feed speed, direction and angle of cutting tool inclination, and select the types of tool path diagrams based on the shape variability and roughness of the surface after machining.

The paper is organized as follows: after the introduction, the second section analyzes the review of literature in the field of milling of free-form surfaces on CNC machine tools. The third section is devoted to the proposed optimization method, of which verification is presented in the case study section (fourth). The research is summarized in the fifth section.

## 2. Milling of Free-Form Surfaces on Multi-Axis Machine Tools

Multi-axis machining of free-form surfaces carried out with a ball milling tool is a very complex process. This is due to complexity and variability of the workpiece shape, kinematics of the cutting process and the shape of the cutting tool. During machining with the use of spherical milling cutters, traces that represent the shape of the used cutting tool are created. Based on previous work [25] for spherical milling cutters, the relationships between: the radius of the spherical part of the tool *R*, the radial cutting depth *a_e_*, the angle of inclination of the surface *γ* and the cusp height *H_c_* can be written as follows (1).

(1) $$a_{e}=\sqrt{8\times R\times H_{c}-4\cdot H_{c}^{2}}\times \cos\left(\gamma \right)$$

The relations given above are valid for the case where *f_z_ ≤ a_e_*; in the opposite case, the radial cutting depth *a_e_* parameter in the above equation should be replaced with feed per tooth *f_z_*. In addition to the geometric representation of the tool shape, the geometric microstructure of the surface is influenced by, among others, deflection and wear of the cutting tool, plastic deformation in the decohesion zone, and geometrical errors of the machine tool. This results in the formation of a geometric microstructure of a surface inferior to that which results from the theoretical relationship. This phenomenon is particularly visible for machining of the hard-to-machine materials like steel in a hardened state, during which large values of the total cutting force components are observed, resulting in large deformations of the cutting tool [26]. In order to limit the values of the cutting force and tool deflection, the tool should be as small as possible and have small outriggers [27]. The usage of the inclination of the cutting tool in the form of a spherical cutter has a positive effect on the geometric structure of the surface. When machining with the ball milling cutters, the effective cutting speed is calculated for the effective tool diameter. Due to the zero effective cutting speed in the tool axis, in order to avoid plastic deformation of the machined layer, tool tilting strategies and machining on the five-axis machine tools are applied [28]. 

With the use of modern CAD/CAM systems, it is possible to design the machining process carried out, whilst maintaining a constant angle of the cutting tool inclination relative to the normal contour of the workpiece fragment. 

**Figure 1:**
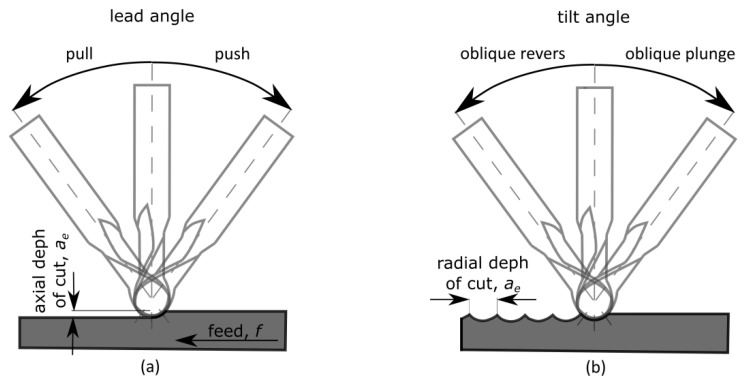
Tool inclination in the feed direction (**a**) and perpendicular to the feed direction (**b**).

The tool inclination is used in the direction of the feed, perpendicular to the direction of feed or both directions at the same time [17]. Figure 1 shows how to define the tool inclination.

The cutting tool tilting causes the complexity of the cutting process kinematics, which makes it possible to cut with [19]:

- push–oblique plunge,
- push–oblique revers,
- pull –oblique plunge,
- pull–oblique revers.

The above encompasses; the direction and value of the inclination angle of the cutting tool influence on the size of a fragment of the cutting edge involved in machining (chipped cutting edge) [20], chip geometry [19], component values of the cutting force [29], as well as microstructure and surface roughness of the machined surface [17]. 

**Figure 2:**
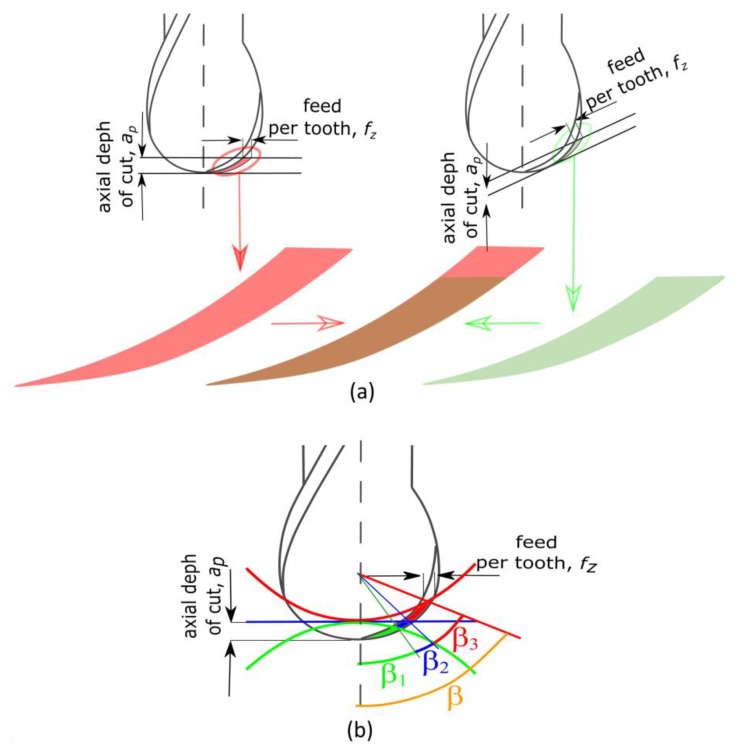
Change of the cutting layer cross-sectional area *A_t_* during machining of an inclined and un-inclined surface (**a**) and surface with different shape (**b**).

When the rounded part of the tool cuts, the change of the tool inclination angle does not affect the shape and surface area of the machined layer [17]. On the other hand, the variable geometry of the machined part influences on the cross-sectional area *A_t_*. As the inclination angle increases, the cross-sectional area of the cutting layer decreases. This is illustrated graphically in Figure 2a.

In analyzing the shape of the machined surface, changes to the cross-section of the cutting layer and the engagement of the tool edge in cutting occur [23]. For the convex surface, the observed cross-section of the cutting layer and the engagement of the cutting edge *β* = *β_1_* assume a value lower than for the concave surface where *β* = *β_1_* + *β_2_* + *β_3_*. These changes are shown in Figure 2b.

## 3. An Algorithm of the Selection of the Cutting Data and Tool Inclination Angle

When machining the free-form surfaces on the multi-axis numerical machine tools, despite the constant cutting parameters, the variable shape of the surface results in the variability of the cross-cut section, which results in variation of the forces and deterioration of the quality and roughness of the machined surface. At the same time, it was found that the analyzed literature concerns only a small scope of the analyzed process. Investigations allow only the selection of constant values of the technological parameters (cutting speed, feed, radial depth of cut and tool inclination), not taking into account the variability of the cross-section of the machined part and the shape of the machined part, occurring during the machining of the free-form surfaces.

In order to additionally take into account the variability of the cross-section of the cutting layer and the shape of the workpiece, an optimization method was developed. The proposed method is based on the thesis: During the free-form surface machining on the multi-axis machine tools using the ball milling cutters, the cross-sectional area changes, and affects the values of the cutting force components, which cause the deformation of the cutting tool and deterioration of the machined surface quality. It is possible, by adjusting the proper tool inclination angle, to minimize the values of the total cutting force components, and then, by modifying the feed rate, to stabilize the values of the total force components and the cross-sectional area of the cutting layer. Stabilization at the assumed level of the cutting force components allows for the control of the cutting tool deflection, which positively affects the quality of the machined surface.

**Figure 3:**
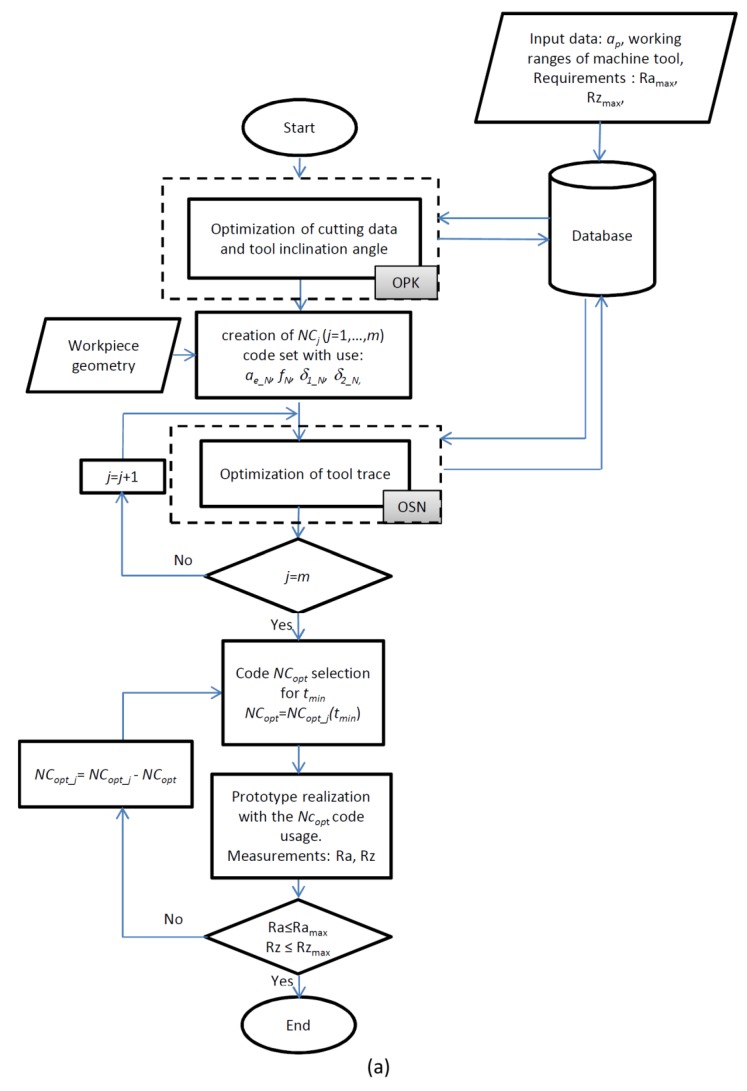

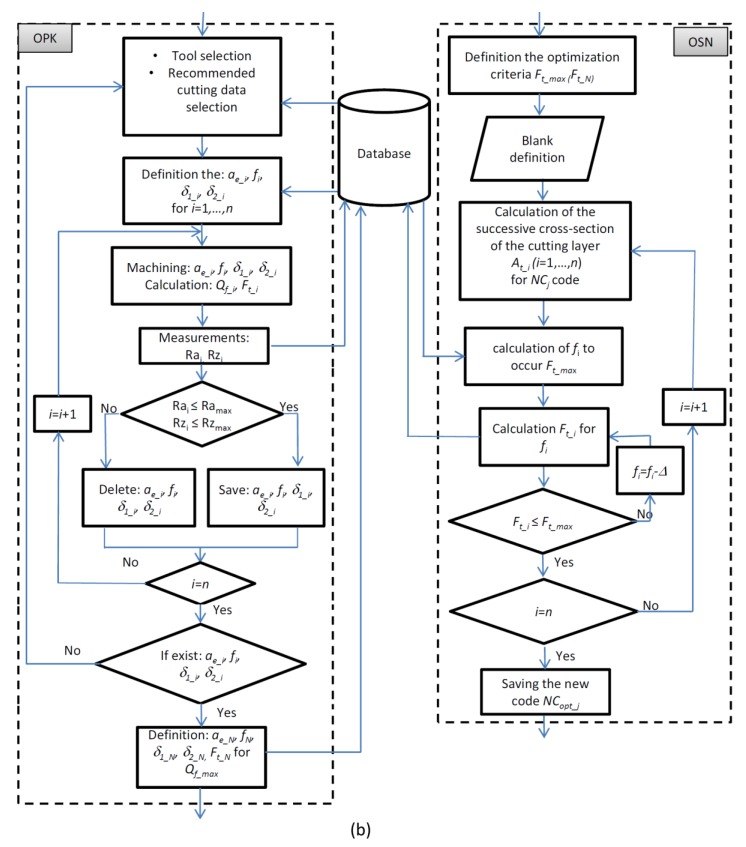
Optimization algorithm: (**a**) main algorithm; (**b**) OPK and OSN stage.

In order to implement the proposed method, an algorithm was developed (Figure 3a,b). The proposed method allows one to modify the feed speed, cutting tool angle and tool path pattern. It was developed mainly to improve the surface quality and increase the manufacturing efficiency of the finishing machining of the molds and matrices.

In the proposed method, the following input data are determined: shape and material of the part, depth of cutting *a_p_*, machine tool parameters (working space, feed and spindle speed ranges) and requirements for the quality of the machined surfaces, which considered the roughness parameters (Ra_max_, Rz_max_) as the limitations of the optimization process. 

The optimization method consists of two stages. In the first stage, an optimization of the machining process (OPK) is carried out to determine the nominal values of the cutting parameters (*a_e_N_*, *f_N_, δ_1_N_*, *δ_2_N_*), enabling the creation of a flat surface with assumed surface roughness parameters (e.g. Ra_max_, Rz_max_) with maximum cutting efficiency *Q_f_max_*. Initially, the ranges of variability of the analyzed cutting parameters are determined: *a_e_i_*, *f_i_*, *δ_1_i_*, *δ_2_i_* for *I* = 1,…,*n*. At this stage, the nominal cross-section of the machined layer is also determined and the efficiency of the machining process *Q_f_i_* = *a_e_i_* · *f_i_* [mm^2^/min] and cutting force components, e.g., *F_t_i_* are calculated. Calculations of the cutting force components are performed using FEM, and they can be optionally replaced by measurements. For the purposes of the machining process optimization, a series of experimental tests should be performed or, if they exist, the available results of the research and the technological base could be used. After machining, the microgeometry measurements of the machined surfaces (e.g. Ra_i_, Rz_i_) are performed. A set of the best parameters is chosen, which fulfills the assumed quality criteria and the highest machining efficiency *Q_f_max_*_._ The nominal force value *F_t_N_* < *F_t_max_* for the machining of a flat surface by using the inclined tool was calculated. If the experimental tests do not permit one to obtain a surface with the assumed parameters, a new set of cutting parameter ranges is defined and optionally, another type of cutting tool is typed and the entire procedure is repeated.

In the next step, the geometry of the workpiece is defined and a set of *NC_j_* codes is created for *j* = 1, …, *m* with cutting parameters *a_e_N_, f_N_,* *δ_1_N_,* *δ_2_N_.* The creation of a series of the *NC_j_* codes is intended to apply various types of tool path diagrams. In the next step, the optimization (OSN algorithm) of the generated *NC_j_* codes is performed.

Optimization aims align the values of the cutting force components and the cross-section of the machined layer by changing the feed rate. It is conducted on the basis of the variation of the tangential component value of the total cutting force *F_t_*, resulting from the variation of the machined surface shape and machined cross-sectional area *A_t_*. FEM is used to calculate the force value changes. In the first stage, based on *F_t_N_*, the optimization criterion *F_t_max_* is defined. The force value *F_t_max_* may be greater than *F_t_N_* to increase the efficiency of the machining process, or less than *F_t_N_* to improve stability. 

Next, the blank is defined, and then a model of the workpiece is discretized to calculate the next cross sections of the cutting layer *A_t_i_* (for *i* = 1, …, *n*), occurring during cutting time with the nominal feed rate *f_N_*. 

The optimization is based on the calculation of the feed value *f_i_*, which will modify the cross-section of the cutting layer *A_t_i_* and the magnitude of the force *F_t_i_*. In the first stage, the feed rate *f* is determined, during which the expected force value occurs as *F_t_i_*=*F_t_max_*. Then, the correctness of the calculations is checked. The force value *F_t_i_* for the new *A_t_i_* and *f_i_* is calculated, on the condition that *F_t_i_* ≤ *F_t_max_* is checked. 

If the criterion is fulfilled, the calculations are made for the next cross-section of the machined layer *A_t_i_* (*i* = *i* + 1). If the condition is not fulfilled, the feed rate is reduced (for example by *Δ* = 2%), which reduces the *F_t_i_* force value, and the correctness of the calculations is checked again.

After completing all calculations (*i* = *n*) for the defined sections of the cutting layer *A_t_i_*, the new *NC_opt_j_* code is saved and the procedure is performed for the next *NC_j_* code (*j* = *j* + 1). After optimizing the entire set of the created codes (*j* = *m*), the *NC_opt_* code is selected from the *NC_opt_j_* codes, the use of which ensures the shortest machining time *t_min_*. The prototype is manufactured and the geometric surface microstructure measurements are performed. If the prototype with the assumed surface quality is made (Ra ≤ Ra_max_, Rz ≤ Rz_max_), the procedure is terminated. If the criterion is not fulfilled, the set of *NC_opt_j_* codes is reduced by the *NC_opt_* code and the procedure is repeated from the place where the *NC_opt_* code is selected. The discussed procedure is presented in the form of algorithms in Figure 3a,b.

## 4. Case Study

### 4.1. Material and Experimental Setup

The machining was carried out on a machine tool DMU Ultrasonic 20 linear. Parts with the flat surfaces with dimensions of 5 × 5 mm made of chromium-molybdenum hot work tool steel (AISI H13) with a hardness of 50 HRC were machined. The chemical composition of this material is shown in Table 1. The mechanical properties of the AISI H13 hot work tool steel are presented in Table 2. In order to verify the proposed method, a free surface formed on the basis of two spline curves was machined. A spherical milling tool with edges made of CBN (catalog designation CBN2XLBR0100N050S04) was selected. The geometry of the cutting tool is presented in Appendix A (Table A1).

Recommended cutting data values for the selected tool and workpiece material are presented in Table 3.

The cutting tests were carried out at the constant cutting speed and axial depth of cut (*v_c_, a_p_*). The radial cutting depth *a_e_*, feed rate *f*, and the inclination angle of the tool in feed *δ_1_*, perpendicular to feed *δ_2_* directions, were changed. Table 4 presents the values and ranges of the cutting parameters’ variation used in the experiments.

During the research, the Taguchi method, based on the L16 orthogonal table for the four input parameters, was used [30]. Cutting cases were analyzed: push–oblique plunge, push–oblique revers, pull–oblique plunge, pull–oblique revers, and therefore, four sets of sixteen attempts were made. Table 5 presents the Taguchi L16 orthogonal table with values of the analyzed cutting parameters.

Using the Taguchi method, the values of S/N (signal to noise) coefficients were determined. The parameters which most strongly influenced the process were sought, and the optimal set of input parameters was selected. This action allowed the selection of the cutting data, the use of which allows the creation of a flat surface with the assumed quality level in the shortest possible time.

The ninth grade of roughness was assumed as a restriction related to the quality of the surface, (Ra_max_ = 0.32 µm, Rz_max_ = 1.6 µm). The measurements of the surface geometric microstructure were made using the profilograph Form TalySurf Intra 50. Measurements were made based on norms ISO 4287, ISO 25178 and EUR 15178N. 

Numerical calculations were applied to predict the values of the total cutting force components. Calculations performed with the use of Finite Element Method have sufficient accuracy to analyze and optimize machining processes [24,31,32].

In the first stage of the algorithm (OPK) the Johnson–Cook constitutive equation (2) was employed to model the plasticity of the workpiece material. The equation defines the plastic flow stress as a function of plastic strain, strain rate and temperature:

(2) $$\sigma =\left(A+B\varepsilon^{n}\right)\left[1+C\ln\left(\frac{\dot{\varepsilon }}{{\dot{\varepsilon }}_{0}}\right)\right]\left[1-{\left(\frac{T-T_{r}}{T-T_{m}}\right)}^{m}\right]$$

Coefficients *A, B, n, C*, and *m* represent the initial yield strength, hardening coefficient, strain hardening exponent, strain rate sensitivity, and thermal softening coefficient.

*ɛ*, *T_r_* and *T_m_* mean, respectively, the reference strain rate, reference temperature and melting temperature of the workpiece. The material constants of the J-C flow stress model for AISI H13 steel are presented in Table 6.

Calculations were performed with the support of the AdvantEdge software [33]. The cutting force measurements could be an alternative to the FEM calculations. 

The cutting force material model applied in the second stage of the algorithm (OSN) uses Equations (3) and (4).

(3) $$\sigma_{n}=BC1\cdot h^{B1}\cdot v_{c}^{A1}-{\left(1-\sin\left(\alpha \right)\right)}^{C1}$$

(4) $$\sigma_{f}=BC2\cdot h^{B2}\cdot v_{c}^{A2}-{\left(1-\sin\left(\alpha \right)\right)}^{C2}$$

where:

*σ_n_,σ_f_*—normal and frictional pressures on the rake face

*h—*average uncut chip thickness

*v_c_*—cutting speed

*α* —rake angle

Values of the material model coefficients are presented in Table 7. Calculations of the cutting forces were performed with the support of the Production Module software [33].

**Figure 4:**
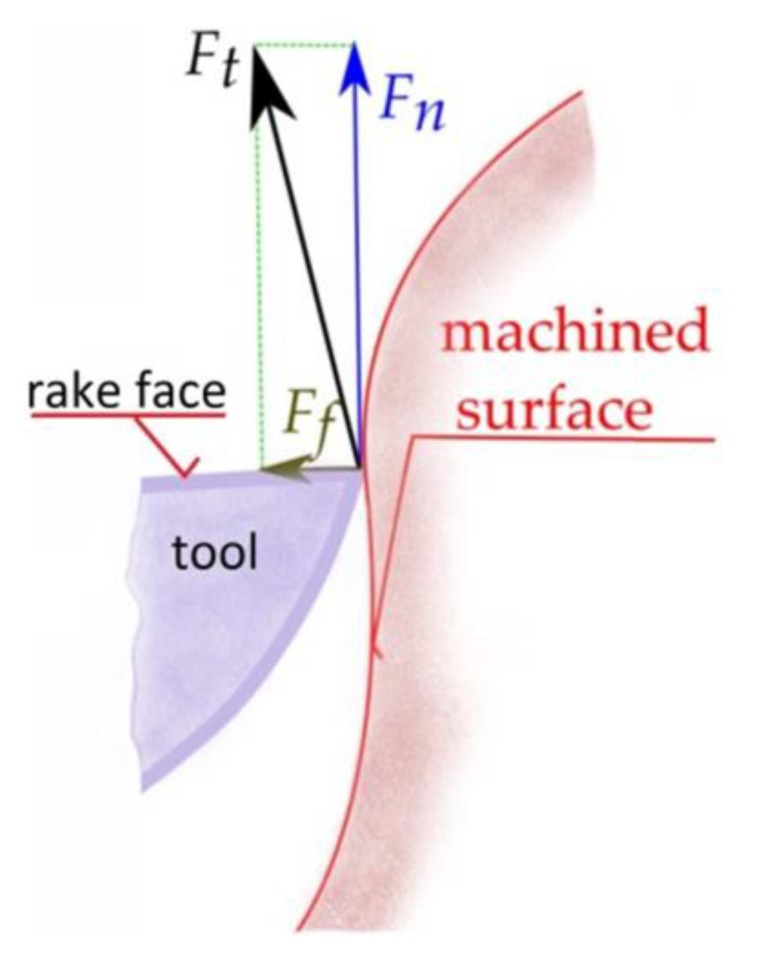
The scheme of the forces acting in the cutting zone.

Knowledge of the stress, acting in the normal and tangential direction to the surface, enables the calculation of the values of the normal and tangential components of the cutting force *F_n_* and *F_f_* (5) acting on the surface *S*, schematically shown in Figure 4.

(5) $$F_{n}=\int_{S}\sigma_{n}\times dS,\;\;=\int_{S}\sigma_{f}\times dS$$

In order to determine the optimal cutting data, the machining efficiency obtained for the analyzed cutting parameters was calculated (6).

(6) $$Q_{f}=a_{e}\cdot f$$

### 4.2. Optimization of the Cutting Data and Inclination Angle of the Tool—Stage I (OPK) 

**Figure 5:**
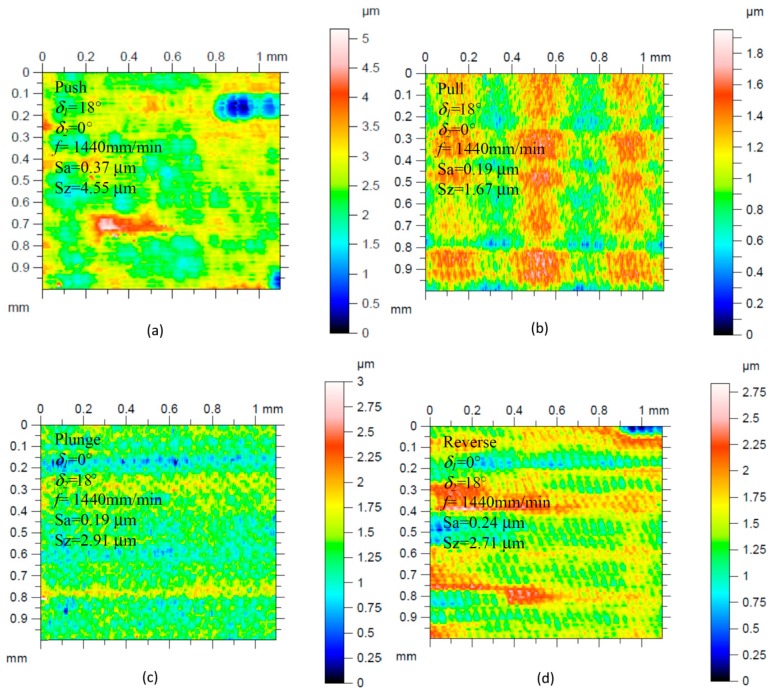
Isometric views of the exemplary surface roughness made with different tool tilt strategies: (**a**) Pusch; (**b**) Pull; (**c**) Plunge; (**d**) Reverse, constant parameters *n* = 27000 rev/min and *a_e_* = 0.025 mm.

As a result of the conducted research, the influence of the analyzed input parameters on the surfaces roughness was observed. Figure 5 shows the views of the exemplary surfaces obtained as a result of using different directions of the cutting tool inclination.

**Figure 6:**
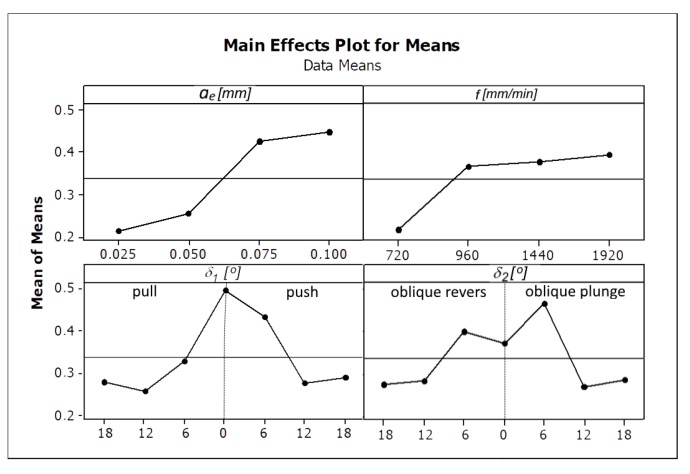
The influence of the analyzed factors on the means values of the surface roughness parameter Ra.

**Figure 7:**
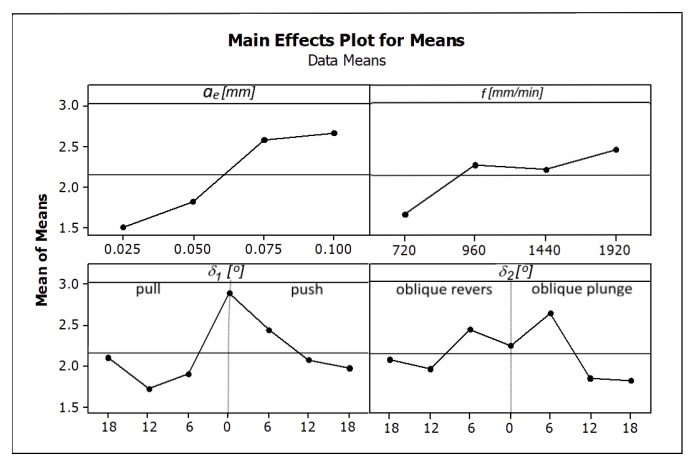
The influence of the analyzed factors on the means values of the surface roughness parameter Rz.

Increasing the feed speed and radial depth of the cut allows the workpiece machining process to work at a higher efficiency *Q_f_*, but not all surfaces are of sufficient quality. Analyzing the influence of the direction and tool inclination angle on the surface roughness, its non-linear character was also observed. The surface quality improves as the value of the tool inclination angle *δ_1_* increases. In the case of the angle *δ_2_*, the tilt above 12° should be used. The influence of the analyzed cutting parameters on the surface roughness parameters Ra and Rz is shown in Figure 6 and Figure 7.

**Figure 8:**
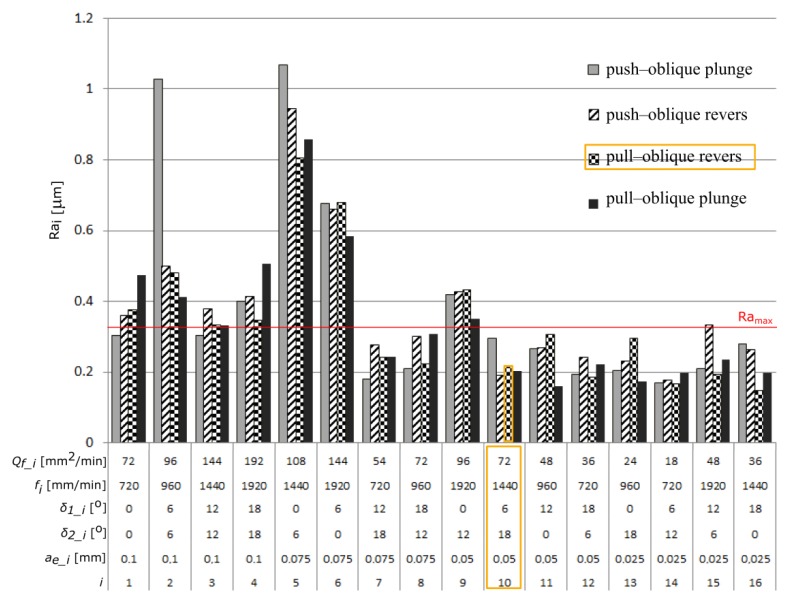
The average values of the surface roughness parameter Ra measured during the tests.

**Figure 9:**
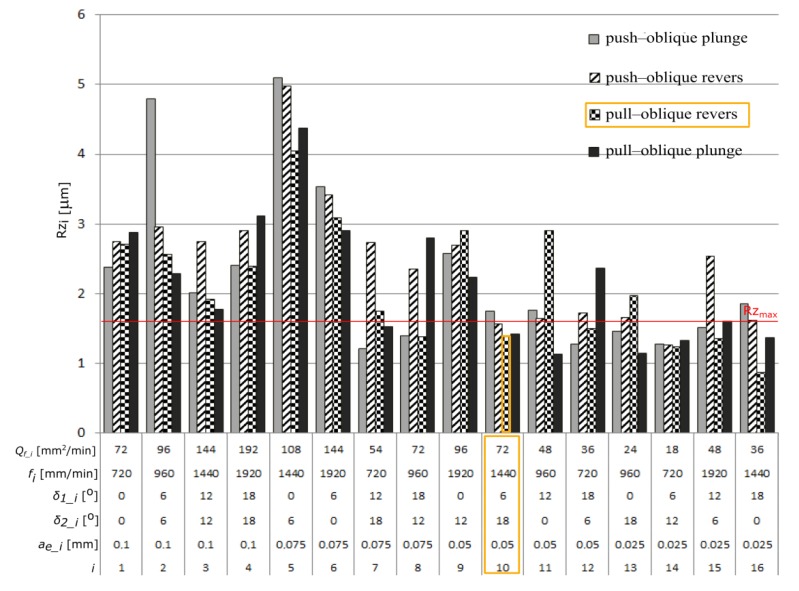
The average values of the surface roughness parameter Rz measured during the tests.

Among the analyzed parameters the radial depth of cut *a_e_* has the biggest influence on the values of the parameters Ra and Rz. The angles *δ_1_* and *δ_2_* and the feed rate *f* have the less impact (Figure 6 and Figure 7) Figure 8 and Figure 9 present the average values of the surface roughness parameters Ra and Rz, measured for the analyzed machining cases. In Appendix B there are Table A2 with average values of the analyzed surface roughness parameters. After analyzing Figure 8 and Figure 9, it is possible to determine the values of the cutting parameters and cutting process kinematics, enabling formation of a flat surface fragment characterized by surface roughness parameters Ra and Rz fulfilling the assumed criterion (Ra_max_ i Rz_max_).

**Figure 10:**
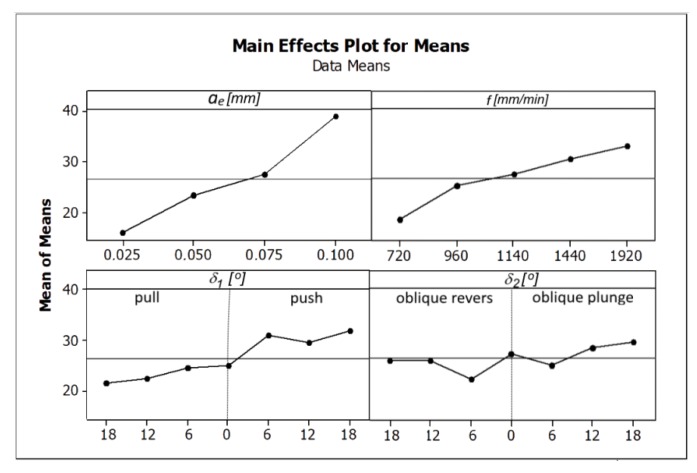
The influence of the analyzed factors on the calculated values of the tangential component (*F_t_*) of the total cutting force.

Figure 10 shows the influence of the analyzed factors on the value of the tangential component of the total cutting force.

**Figure 11:**
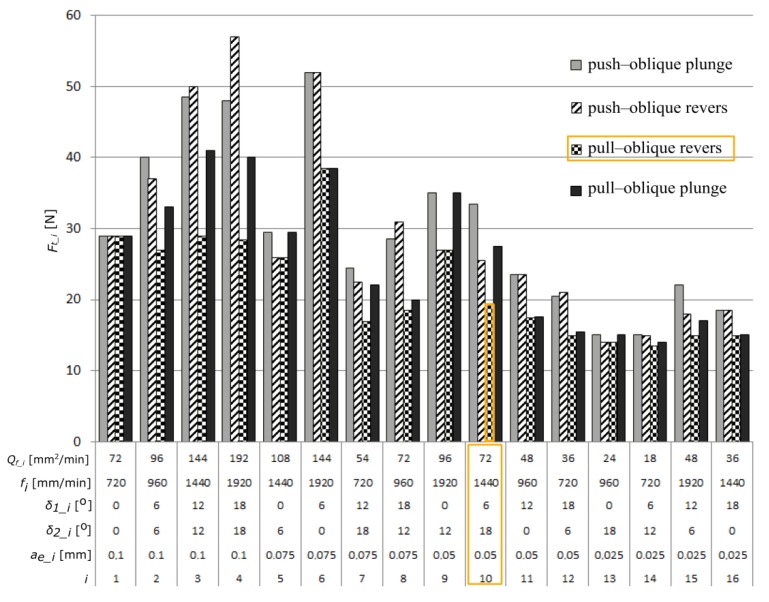
The average values of the tangential component of the total cutting force *F_t_*, calculated in the analyzed machining cases.

Among the analyzed parameters, the radial depth of cut has the biggest influence on the values of the total cutting force components. The feed rate *f*, and angles *δ_1_* and *δ_2_* have the least amount of impact. Figure 11 presents the average value of the tangential component of the total cutting force calculated for the analyzed machining cases, obtained by changing the angle and direction of the tool inclination.

As a result of the research, a number of surfaces characterized by the assumed surface roughness were obtained. The use of the analyzed parameters allows the obtainment of a surface with assumed geometrical parameters at the maximum efficiency of the machining process *Q_f_*, at the level of 72 mm^2^/min. As the optimal cutting parameters, two sets, no. 8 and 10, were selected. For further analysis, due to the more favorable case of the tool tilt (*δ_1_10_* = 6° *<* *δ_1_8_* = 18°), set no. 10, nominal cutting data (*f_N_* = 1440 mm/min, *δ_1_N_* = 6°, *δ_2_N_* = 18°, *a_e_N_* = 0.05 mm), and pull–oblique revers cutting process kinematics were selected. The nominal value of the total cutting force component was also selected *F_t_N_* = 19.5 N.

### 4.3. Selection of the Machining Strategy and Optimization of the Feed Speed—Stage II (OSN)

**Figure 12:**
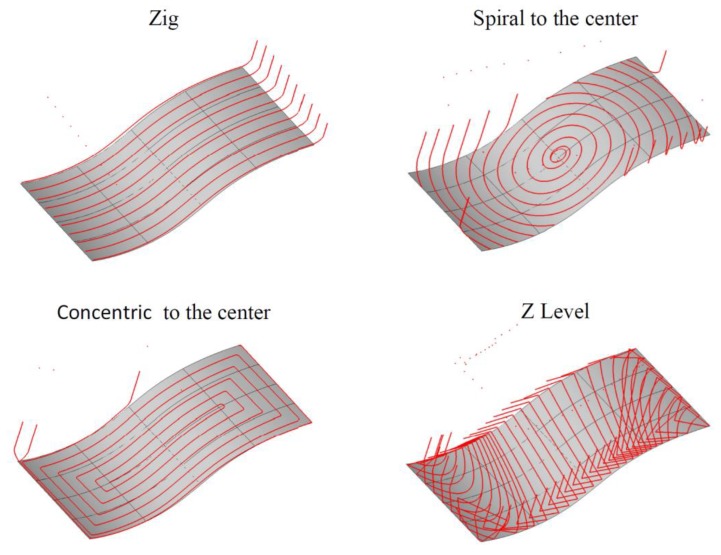
Simplified views of the created tool paths.

For the purpose of implementing the second stage of the proposed method, the area created on the basis of two spline curves was defined. Next, a set of tool paths was generated using the CAM software. The created tool paths are shown schematically in Figure 12.

In the next step, the working lengths of the tool paths, the length of the setting movements and the machining times for the selected nominal cutting data (*f_N_* = 1440 mm/min, *δ_1_N_* = 6°, *δ_2_N_* = 18°, *a_e_N_* = 0.05 mm) and pull–oblique revers cutting process kinematics were calculated.

In the next step, the values of the total cutting force components were calculated and the feed rate optimization was made. As the optimization criterion, the value of the tangential component of the total cutting force was set to *F_t_max_* = 20 N.

**Figure 13:**
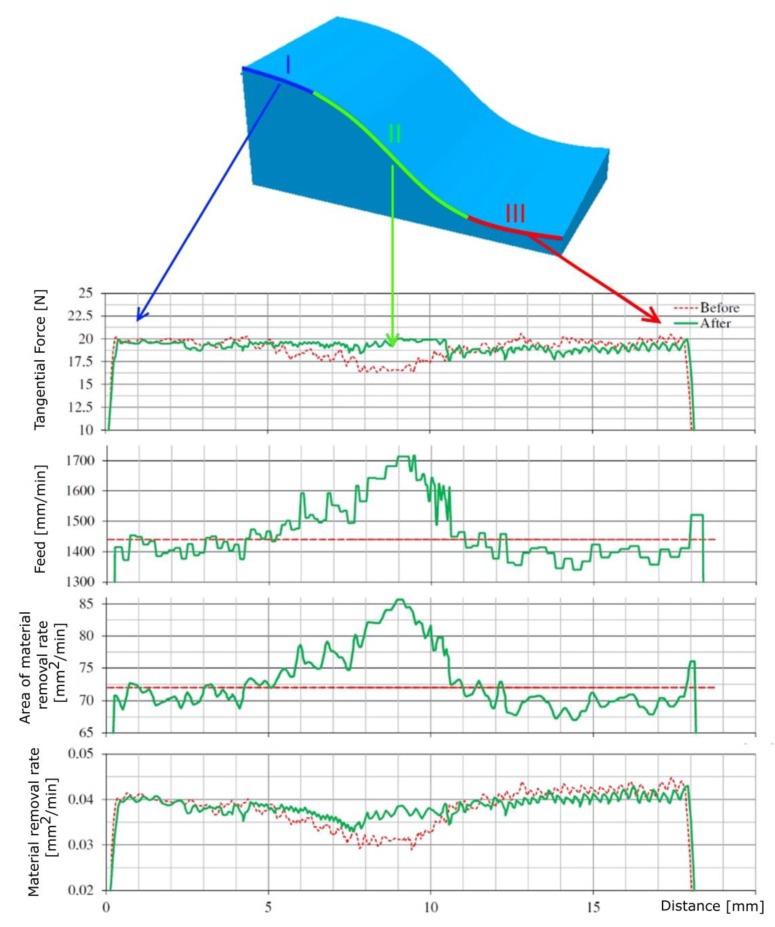
Courses of the tangential component of the total cutting force and the change of the feed rate and machining process efficiency obtained for Stage II.

Figure 13 shows the courses of the tangential cutting force component, feed rate and machining process efficiency calculated during a single tool pass along the tool path for the optimized (After) and non-optimized (Before) feed rate. In Figure 13 (shown), the operation of the proposed optimization method can be seen. In the initial stage (area I), a surface with an outline similar to a straight line is machined. The cross-sectional area of the machined layer *A_t_* and the values of the components of the total cutting force are equal to the nominal value, therefore the feed rate is set close to the nominal range. In the next machining step (area II), the inclined surface is machined with the outline of a convex arch. At this stage, during cutting with the nominal feed speed, the values of the cutting forces and the cut of the cutting layer decrease. In order to stabilize the values of the components of the total cutting force, the cross-sectional area of the machined layer is increased by increasing the feed rate. In the final area (area III), a surface with an outline of a concave arch is made.

During machining with the nominal feed speed, the different values of the cutting forces and the cross-cut section are observed. As a result of the optimization, the feed rate is reduced, in order to stabilize the value of the components of the total cutting force.

The optimization process was performed for all created tool paths. Table 8 summarizes the values of the tool path lengths and machining times, using them for the optimized and non-optimized feed rate value.

### 4.4. Verification—Machining of the Element on the Machine Tool and the Surface Roughness Measurements

**Figure 14:**
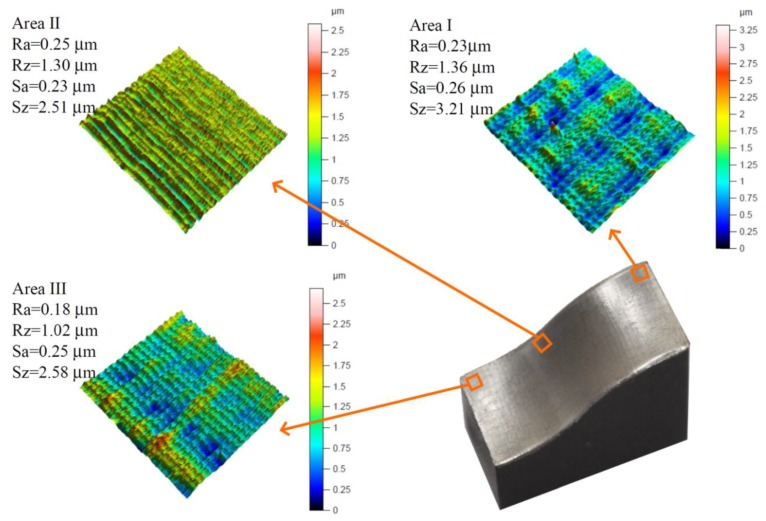
Isometric views of the machined surface using the proposed method.

In order to verify the proposed method, the selected surface was machined. The *NC_opt_* code, constant values of the cutting parameters (*δ _1_N_* = 6°, *δ_2_N_* = 18°, *a_e_N_* = 0.05 mm), pull–oblique revers cutting process kinematics, variable feed rate and concentric machining strategy were used for machining. Then, measurements of the geometric structure of the surface were made and surface roughness parameters were determined. Figure 14 shows the isometric views of the surface for areas I, II, III and the values of the measured roughness parameters. 

The roughness parameter values and the isometric views of the fragments of the surface shown in Figure 14 proved the correctness of the proposed method. The surface roughness parameters were stabilized, within the 9 class of the surface roughness, independently of the surface angle inclination.

## 5. Conclusions

This paper proposes a comprehensive method of the optimization of the cutting data and tool path pattern for finishing the free-form surfaces made of the steel in a hardened state. At the first stage of the method (OPK), an increase (by 9%) of the surface efficiency of the machining process was obtained: from 66.5 mm^2^/min using the cutting parameters recommended by the manufacturer (*f* = 1900 mm/min, *δ_1_* = 0°, *δ_2_* = 10°, *a_e_* = 0.035 mm), to 72 mm^2^/min for the selected cutting parameters (*f_N_* = 1440 mm/min, *δ_1_N_* = 6°, *δ_2_N_*= 18°, *a_e_N_* = 0.05 mm) and pull–oblique revers cutting process kinematics. In the second part (OSN), during the optimization process of the concentric tool path, an additional 15% increase in the machining process efficiency and the required roughness of the machined surface were obtained.

The results of the research showed the correctness of the thesis, that changing the direction and angle value of the cutting tool affects the value of the total cutting force components. During the machining of the free-form surfaces, there are changes in the cross-section of the machined layer, depending on the local shape of the workpiece. By modifying the feed rate, it is possible to control the cross-section of the cutting layer and the values of the total cutting force components. Their stabilization at the assumed level aligns the analyzed surface roughness parameters, which positively affects the quality of the machined surface. The method and calculations proposed in the manuscript were verified by creating a surface characterized by the assumed surface roughness, as described in Section 4.4.

During the research, the following conclusions were also noticed:

- The significant, non-linear influence of the analyzed inclination angles of the cutting tool and cutting process kinematics on the machined surface roughness is observed.
- Increasing cutting force results in increased load and bending of the cutting tool, which causes deterioration of the surface roughness.
- The radial depth of cut *a_e_* has the greatest impact on the surface roughness parameters. It shows that the influence of the geometric representation of the cutting tool shape is dominant over the physical phenomena occurring in the cutting zone.

The method proposed in the work assumes that during machining there is no wear of the tool, which has an impact on the surface roughness. During the tests, no significant tool wear was observed, but when machining larger surfaces, the model should consider the effect of cutting edge wear on surface roughness.

## Figures and Tables

**Table 1:** Chemical composition of the hot work tool steel (AISI H13), (%).

C	Mn	Si	Cr	Mo	V
0.4	0.4	1.0	5.25	1.35	1.0

**Table 2:** Mechanical properties of the hot work tool steel (AISI H13).

Tensile Strength [MPa]	Thermal Conductivity [W/m°C]	Density [Kg/m^3^]	Modulus of Elasticity [GPa]	Poisson Ratio
1200	17.6	7750	215	0.27

**Table 3:** Recommended cutting data values.

*n* [rev/min]	*v_c_* [m/min]	*a_p_* [mm]	*a_e_* [mm]	*f* [mm/min]	*δ_1_* [°]	*δ_2_* [°]
27000	150	0.1	0.035	1900	0	10

**Table 4:** The range of variation and the values of the adopted cutting process data.

*n* [rev/min]	*v_c_* [m/min]	*a_p_* [mm]	*a_e_* [mm]	*f* [mm/min]	*δ_1_* [°]	*δ_2_* [°]
27000	150	0.1	0.025–0.1	720–1920	0–18	0–18

**Table 5:** Taguchi L16 orthogonal table with values of analyzed parameters.

Lp.	A	B	C	D	*a_e_*	*δ_1_*	*δ_2_*	*f*
					[mm]	[°]	[°]	[mm/min]
1	4	1	1	1	0.1	0	0	720
2	4	2	2	2	0.1	6	6	960
3	4	3	3	3	0.1	12	12	1440
4	4	4	4	4	0.1	18	18	1920
5	3	1	2	3	0.075	0	6	1440
6	3	2	1	4	0.075	6	0	1920
7	3	3	4	1	0.075	12	18	720
8	3	4	3	2	0.075	18	12	960
9	2	1	3	4	0.05	0	12	1920
10	2	2	4	3	0.05	6	18	1440
11	2	3	1	2	0.05	12	0	960
12	2	4	2	1	0.05	18	6	720
13	1	1	4	2	0.025	0	18	960
14	1	2	3	1	0.025	6	12	720
15	1	3	2	4	0.025	12	6	1920
16	1	4	1	3	0.025	18	0	1440

**Table 6:** Johnson–Cook constitutive model parameters for AISI H13 steel [32].

*A* MPa	*B* MPa	*n*	*C*	*m*	ɛ
1469	321.39	0.278	0.028	1.18	1.0

**Table 7:** Values of the material model coefficients.

*BC*1	*B*1	*A*1	*C*1	*BC*2	*B*2	*A*2	*C*2
4030	0.0058	0	0.6153	0.6021	−0.0031	0	−2.2125

**Table 8:** List of tool path lengths and machining times using optimized and non-optimized NC code.

	The Length of the Working Movement	The length of the Setting Movement	Time for Non-optimized Feed Rate	Time for Optimized Feed Rate
	[mm]	[mm]	[s]	[s]
Zig	4706	5658	217	190
Spiral	5030	5320	238	208
Concentric	4144	181	183	155
Z Level	4883	5762	285	204

## Nomenclature

*f, f_N_*	Feed speed, Nominal value of feed speed enabling the milling of a flat surface with assumed surface roughness
*f_z_*	Feed per tooth
*a_p_*	Axial depth of cut
*a_e_, a_e_N_*	Radial depth of cut, Nominal value of radial depth of cut enabling the milling of a flat surface with assumed surface roughness
*δ_1,_ δ_1_N_*	Tool inclination angle in the feed direction (lead angle), Nominal lead angle enabling the milling of a flat surface with assumed surface roughness
*δ_2_, δ_2_N_*	Tool inclination angle perpendicular to the feed direction (tilt angle), Nominal tilt angle enabling the milling of a flat surface with assumed surface roughness
*v_c_*	Cutting speed
*n*	Spindle Speed
*R*	Radius of the spherical part of the tool
*A_t_*	Machined cross-sectional area
*β*	Cutting edge engagement angle
*γ*	Surface inclination angle
*Ra, Ra_max_*	Arithmetical mean deviation of the assessed profile, Maximum value of Ra parameters used as the limitations of the process optimization
*Rz, Rz_max_*	Average distance between the highest peak and lowest valley, Maximum value of Rz parameters used as the limitations of the process optimization
*Sa*	Average Roughness evaluated over the 3D area
*Sz*	Sum of the largest peak height value and the largest pit depth value evaluated over the 3D area
*H_c_*	Cusp height
*Q_f_, Q_f_ max_*	Cutting efficiency, Maximum cutting efficiency obtained while milling with nominal cutting data
*NC, NC_opt_*	NC code, Optimized NC code
*F_t_, F_t_N_, F_t_max_*	Tangential force component (tangential to the machined surface), Nominal value of *F_t_*, Maximum value of *F_t_* used as the limitations of the process optimization
*F_f_*	Friction force (component of the cutting force, tangential to the rake face)
*F_n_*	Normal force (component of the cutting force, normal to the rake face)
*σ_f_*	Frictional pressures on the rake face
*σ_n_*	Normal pressures on the rake face
*h*	Average uncut chip thickness
*α*	Rake angle of the tool
*S*	Contact surface of chip with the rake face of the tool
*t, t_min_*	Time, Time of machining with NC_opt_ code

## Appendix A

**Table A1:** Geometry of the milling tool.

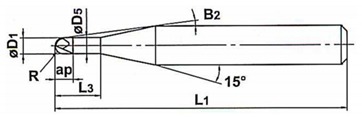

***L_1_*** [mm]	*D_1_* [mm]	*a_p_* [mm]	*L_3_* [mm]	*D_5_* [mm]	*B_2_* [°]	*L_1_* [mm]	*D_4_* [mm]	*z* [-]
1	2	1.5	5	1.9	7.3	52	4	2

## Appendix B

**Table A2:** Average values of the parameters Ra and Rz.

					Push–oblique Plunge		Push–oblique Revers		Pull–oblique Revers		Pull–oblique Plunge	
Lp.	*a_e_* [mm]	*δ_1_* [°]	*δ_2_* [°]	*f* [mm/min]	Ra [µm]	Rz [µm]	Ra [µm]	Rz [µm]	Ra [µm]	Rz [µm]	Ra [µm]	Rz [µm]
1	0.1	0	0	720	0.30	2.38	0.36	2.75	0.38	2.72	0.47	2.88
2	0.1	6	6	960	1.03	4.79	0.50	2.96	0.48	2.57	0.41	2.29
3	0.1	12	12	1440	0.30	2.01	0.38	2.75	0.33	1.93	0.33	1.78
4	0.1	18	18	1920	0.40	2.41	0.41	2.91	0.34	2.40	0.51	3.11
5	0.075	0	6	1440	1.07	5.10	0.95	4.98	0.80	4.05	0.86	4.38
6	0.075	6	0	1920	0.68	3.54	0.66	3.42	0.68	3.09	0.58	2.90
7	0.075	12	18	720	0.18	1.21	0.28	2.73	0.24	1.75	0.24	1.53
8	0.075	18	12	960	0.21	1.39	0.30	2.35	0.22	1.38	0.31	2.80
9	0.05	0	12	1920	0.42	2.57	0.43	2.70	0.43	2.91	0.35	2.23
10	0.05	6	18	1440	0.30	1.75	0.19	1.57	0.22	1.39	0.20	1.42
11	0.05	12	0	960	0.27	1.77	0.27	1.65	0.31	2.91	0.16	1.14
12	0.05	18	6	720	0.19	1.28	0.24	1.73	0.18	1.51	0.22	2.37
13	0.025	0	18	960	0.20	1.46	0.23	1.66	0.29	1.97	0.17	1.14
14	0.025	6	12	720	0.17	1.28	0.18	1.26	0.16	1.24	0.20	1.33
15	0.025	12	6	1920	0.21	1.51	0.33	2.54	0.19	1.36	0.23	1.61
16	0.025	18	0	1440	0.27	1.74	0.27	1.74	0.17	1.12	0.17	1.12
